# End-to-End Estimation of Hand- and Wrist Forces From Raw Intramuscular EMG Signals Using LSTM Networks

**DOI:** 10.3389/fnins.2021.777329

**Published:** 2021-11-17

**Authors:** Alexander E. Olsson, Nebojša Malešević, Anders Björkman, Christian Antfolk

**Affiliations:** ^1^Department of Biomedical Engineering, Faculty of Engineering, Lund University, Lund, Sweden; ^2^Department of Hand Surgery, Sahlgrenska University Hospital, Institute of Clinical Sciences, University of Gothenburg, Sahlgrenska Academy, Gothenburg, Sweden; ^3^Department of Hand Surgery, Skåne University Hospital, Malmö, Sweden; ^4^Department of Translational Medicine, Lund University, Lund, Sweden

**Keywords:** iEMG, force, deep learning, LSTM, recurrent neural networks, regression, proportional control, simultaneous control

## Abstract

Processing myoelectrical activity in the forearm has for long been considered a promising framework to allow transradial amputees to control motorized prostheses. In spite of expectations, contemporary muscle–computer interfaces built for this purpose typically fail to satisfy one or more important desiderata, such as accuracy, robustness, and/or naturalness of control, in part due to difficulties in acquiring high-quality signals continuously outside laboratory conditions. In light of such problems, surgically implanted electrodes have been made a viable option that allows for long-term acquisition of intramuscular electromyography (iEMG) measurements of spatially precise origin. As it stands, the question of how information embedded in such signals is best extracted and combined across multiple channels remains open. This study presents and evaluates an approach to this end that uses deep neural networks based on the Long Short-Term Memory (LSTMs) architecture to regress forces exerted by multiple degrees of freedom (DoFs) from multichannel iEMG. Three deep learning models, representing three distinct regression strategies, were evaluated: (I) One-to-One, wherein each DoF is separately estimated by an LSTM model processing a single iEMG channel, (II) All-to-One, wherein each DoF is separately estimated by an LSTM model processing all iEMG channels, and (III) All-to-All, wherein a single LSTM model with access to all iEMG channels estimates all DoFs simultaneously. All models operate on raw iEMG, with no preliminary feature extraction required. When evaluated on a dataset comprising six iEMG channels with concurrent force measurements acquired from 14 subjects, all LSTM strategies were found to significantly outperform a baseline feature-based linear control regression method. This finding indicates that recurrent neural networks can learn to transform raw forearm iEMG signals directly into representations that correlate with forces exerted at the level of the hand to a greater degree than simple features do. Furthermore, the All-to-All and All-to-One strategies were found to exhibit better performance than the One-to-One strategy. This finding suggests that, in spite of the spatially local nature of signals, iEMG from muscles not directly actuating the relevant DoF can provide contextual information that aid in decoding motor intent.

## Introduction

Voluntary movements of fingers are controlled by intrinsic muscles located in the hand and extrinsic muscles, that also control wrist movement, located in the forearm (Blana et al., 2017). After a wrist disarticulation or transradial amputation, whereas the hand itself is lost, the extrinsic muscles, although shortened, will largely remain in place and innervated. Typically, such remnant muscles can still be contracted voluntarily by the amputee and thereby produce detectable myoelectric activity that can be measured with electromyography (EMG). For many decades now (Wirta et al., 1978), processing EMG signals originating from remnant muscles has been considered a leading candidate in the ongoing pursuit of tools that allow amputees to better control prosthetic hands.

The standard form of myoelectric control interface in commercially available upper limb prostheses is known as *direct control* (Paciga et al., 1980). In this framework, pairs of surface electrodes measure the amplitudes of EMG from antagonistic muscle pairs located superficially in the residual limb; the difference between each pair of channels can subsequently be used to control a single degree of freedom (DoF) of an active prosthesis. Although simple and robust, direct control exhibits some crucial disadvantages compared to the functionality afforded effortlessly by the healthy human hand: First, control is not intuitive, as the activation pattern required to perform a movement do not correspond to the physiologically natural pattern. Second, the limited number of electrode pairs that can be accommodated by the approach means that only a handful of DoFs can be controlled simultaneously, hampering dexterity. This is in sharp contrast to the impressive mechanical abilities of contemporary high-end active hand prostheses—such devices could, if provided with sufficiently precise control commands, articulate a large number of DoFs simultaneously and independently (Saikia et al., 2016). This shortcoming of control has been conjectured to be one of the main drivers of the high abandonment rate of myoelectric upper limb prostheses (Biddiss and Chau, 2007).

A somewhat more recent development is myoelectric control based on *pattern recognition* (Hudgins et al., 1993; Scheme and Englehart, 2011; Zecca et al., 2017). In this control framework, the movement intent of the user is inferred by a machine learning model that operates on continuously segmented multichannel surface EMG (sEMG) signals. To learn an appropriate mapping via supervised learning, training data must be provided to the model in the form of example sEMG time windows and corresponding measures of movement intent (e.g., kinematic regressands or categorical target motion classes). Aside from this requirement of initial calibration data, pattern recognition control exhibits many promising advantages compared to the direct control paradigm: the subjective sensation of control can be made completely intuitive, and a relatively large set of DoFs (dependent on the size of the electrode array) can in theory be controlled simultaneously (Scheme and Englehart, 2011). However, due to problems of robustness and stability over time of algorithms, clinical adoption remains uncommon (Jiang et al., 2012). Furthermore, being predicated on sEMG puts practical limits on the kind of information that can be made available to machine learning models of this kind. Signals originating from deeply situated muscles are attenuated to a significant degree, and even superficial muscles can generate levels of crosstalk that hamper the task of separately decoding multiple DoFs (Lowery et al., 2002). Thus, in addition to improving algorithms, a promising avenue for improving control interfaces is to provide algorithms with more informative raw input signals.

By invasively inserting needle- or fine-wire electrodes directly into muscles, EMG signals that have very precise spatial origin can be acquired. Intramuscular EMG (iEMG) signals of this kind exhibit negligible crosstalk and a high degree of correlation with concurrent kinematics (Lowery et al., 2006; Kamavuako et al., 2009), but would most likely be too delicate to use as the basis of control for a wearable system. Surgically implanted electrodes (Brånemark et al., 2001; Hobby et al., 2001; Kuiken et al., 2009) have been proposed as a way to circumvent this problem and are quickly becoming a realistic alternative fit for widespread adoption. With these approaches, individual muscles can be recorded for extensive periods of time, even chronically, thereby potentially providing a long-lived control interface between the user and the prostheses. This kind of setup entails a further benefit of better resisting electrode shift—a phenomenon known to severely impair the performance of pattern recognition control based on sEMG over time due to distributional shifts (Kyranou et al., 2018).

Only a handful of previous studies (Hargrove et al., 2007; Cipriani et al., 2014; Smith et al., 2014, 2016) have experimentally investigated the use of iEMG as the input to prostheses control interfaces, likely in part due to the inherent difficulties in acquiring iEMG signals invasively. Due to the already highly informative content of iEMG signals w.r.t. concurrent kinematics, such studies have justifiably either opted for the use of dual-site, amplitude-proportional linear control (e.g., Smith et al., 2014), or relatively simple pattern recognition algorithms operating on extracted signal features (e.g., Hargrove et al., 2007). Even so, we hypothesize that more sophisticated signal processing could increase the correlation between intramuscular signals and kinematics further, and, in accordance, a more elaborate approach is examined in the current study. Inspired by the success of deep learning for decoding motor intent from sEMG (for reviews of the field, see (Phinyomark and Scheme, 2018; Rim et al., 2020; Buongiorno et al., 2021; Xiong et al., 2021), we use models based on the Long Short-Term Memory (LSTM) architecture (Hochreiter and Schmidhuber, 1997) to regress forces exerted by multiple DoFs at the level of the hand and wrist from multichannel iEMG acquired from extrinsic muscles. Notably, the proposed algorithms evaluated here differ from existing attempts of iEMG force regression in that no feature extraction step is undertaken; rather, the concurrently measured force values are inferred directly, in an end-to-end fashion, from time windows of minimally preprocessed iEMG voltages. Furthermore, to quantify the impact that combining information originating from different muscles has on force regression performance, we let some models operate on a single iEMG channel and some models operate on all channels in order to automatically learn ways to aggregate spatially encoded information. Whereas the algorithms of the current study were trained and tested offline on a publicly available database, steps were taken to ensure that all parts of the processing pipeline can be executed in a functionally equivalent manner in a real-time scenario.

## Materials and Methods

### Data Acquisition

The database of fine-wire iEMG recordings and concurrent force measurements used in this study was collected for a previous study and has been made publicly available (Malesevic et al., 2020). For the sake of completeness, all properties of the database relevant for the current study are restated in brief here. Data were collected from 14 male, neurologically intact subjects aged between 25 and 57 years (mean age 39 years). All subjects gave informed and written consent prior to participation, and the study was approved by the Regional Ethics Review Board in Lund, Sweden (Dnr 2017-297). Each recording session lasted approximately 30 min and used one out of two possible electrode placement protocols: (I) The Short Residual Limb protocol, targeting the following six muscles of the forearm: flexor carpi radialis (FCR), extensor carpi radialis (ECR), pronator teres (PT), flexor digitorum profundus (FDP), extensor digitorum communis (EDC), and abductor pollicis longus (APL) and (II) The Long Residual Limb protocol, targeting the following six muscles of the forearm: flexor digitorum profundus (FDP), extensor digitorum communis (EDC), abductor pollicis longus (APL), flexor pollicis longus (FPL), extensor pollicis longus (EPL), and extensor indicis proprius (EIP).

Intramuscular EMG (iEMG) signals were sampled at a rate of *F*_*s*_ = 10240 Hz. A custom-built measurement device (Malešević et al., 2019) was used to hold the hand stationary for the duration of acquisition (thus ensuring isometric contractions, as would be the case with forearm amputees) and record forces exerted at the level of the hand and wrist. In total, nine force gauges, corresponding to the major degrees of freedom (DoFs) of the hand and wrist, were used: one per finger (D2–D5), two for the thumb, and three for the wrist. Consequently, each session always comprised six channels of iEMG and nine channels of force (synchronized sample-wise). Two of the subjects carried out both protocols, resulting in a dataset comprising 16 recording sessions, out of which eight were recorded with the Short Residual Limb protocol and eight were recorded with the Long Residual Limb protocol. Subjects were assigned to placement protocols randomly.

In total, each recording session comprised 22 unique *tasks*, each corresponding to a movement incorporating either activation of a single or activation of a combination of some or all of the six muscles being assessed in the study. The current study makes use of only the first eight tasks of the database (shown in Table 1), representing movements that incorporate a single DoF at a time. Furthermore, to not bias training and test data toward higher force levels and to consequently better simulate the intended use case of prosthesis control, only signals originating from the *sine tracking* substage were used. During this substage, the subject was instructed to contract relevant muscles to track a low-frequency (0.1 Hz) sinusoid with amplitude equal to 20% of the force measured during maximum voluntary contraction plotted in real time on a screen. Each of the eight relevant sine tracking substages comprises 10 periods of such a sinusoid.

**TABLE 1:** The subset of movement tasks utilized in the current study.

**Code in database**	**Description**
1.X	Index finger: flexion-extension
2.X	Middle finger: flexion-extension
3.X	Ring finger: flexion-extension
4.X	Little finger: flexion-extension
5.X	Thumb: flexion-extension
6.X	Thumb: adduction-abduction
7.X	Wrist: flexion-extension
8.X	Wrist: supination-pronation

### Preprocessing

As all algorithms proposed in the current study are intended for online execution, signal preprocessing steps were conducted in a manner compatible with this requirement. To initially reduce the impact of voltage spikes and other outlier samples, all iEMG channels were individually clipped at the 99 and 1 percentile levels and subsequently filtered using a second-order digital Butterworth low-pass filter with a cutoff frequency of 500 Hz. Similarly, all force channels were individually low-pass filtered using a second-order Butterworth filter with a cutoff frequency of 10 Hz. Following these introductory preprocessing steps, all signals were downsampled by a factor of 10 (i.e., to *F*_*s*_ = 1024 Hz) in order to reduce computational complexity and facilitate faster convergence of learning algorithms.

Based on iEMG-movement-force matchings provided together with the database, filtered iEMG- and force signals were restructured in the following way: first, each of the nine force channels was separated, on the basis of the sign of the *Tracking Cue* variable provided in the database, into two new channels—one new channel representing positive phase (flexion, adduction, and pronation) and one new channel representing negative phase (extension, abduction, and supination). All 18 resulting phase-specific force channels were subsequently rectified, ensuring that both phases had a positive and similar envelope. Second, each iEMG channel was paired with a single such force channel on the basis of the matchings provided with the database. All force channels that had not been paired with an iEMG channel, i.e., force channels originating from DoFs not actuated by any of the electrode-penetrated muscles, were at this stage discarded. Third, a list of elicited movements (i.e., tasks) that were matched with any of the remaining iEMG-force matchings was created; signal parts originating from elicited movements not contained in the list were discarded and not used further in the current study. As a result, the *T* time samples remaining following this selection could be represented as two synchronous signal matrices with matched rows: **E** ∈ *ℝ*^*T*×6^, containing the six iEMG channels, and **F** ∈ *ℝ*^*T*×6^, containing the six matched force channels. Subject-specific elicited movements remaining after this stage are presented in Table 2.

**TABLE 2:** Overview of subject-wise elicited movements selected for regression model training and testing.

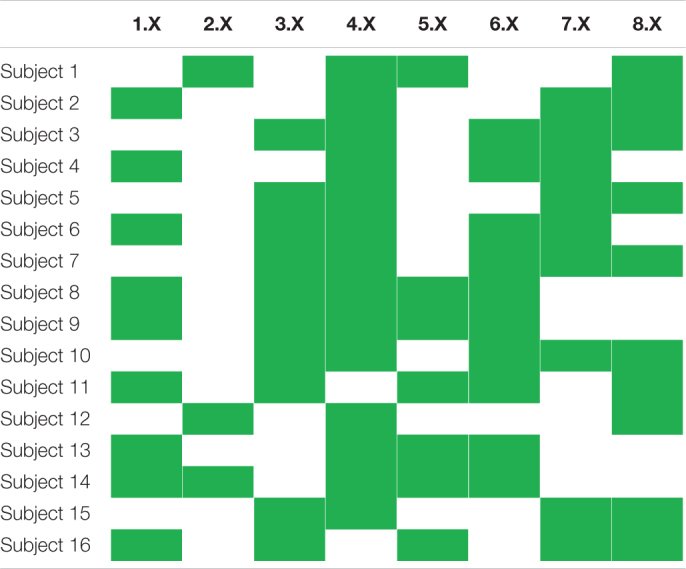

*Cells shaded green represent elicited movements that remained following preprocessing.*

In order to represent the resulting data in a way amenable to machine learning methodology, the iEMG signals contained in **E** were segmented into individual regression instances by using a sliding window of width 512 samples (500 ms) with increments of 64 samples (62.5 ms). Each time window was assigned a ground-truth force vector by simply selecting the last row (i.e., time sample) of the sliding window in **F**. In an online application, this would correspond to inferring the current force from the preceding 500 ms of iEMG, with delays between consecutive inferences of 62.5 ms—well in line with acceptable values of myocontrol delay (Farrell and Weir, 2007).

Intramuscular EMG (iEMG) time windows with appertaining force vectors were lastly partitioned into a training set and a test set on the basis of sinusoid period: signal windows originating from the first 7 periods (out of the available 10) of each sine tracking task were designated as training data, signal windows originating from the 8th period were designated validation data, and signal windows originating from the 9th and 10th period were designated as test data. All iEMG windows were linearly rescaled using the channel-wise mean and standard deviation computed from the training set; training iEMG data thus had zero mean and unit variance. All target force values were similarly normalized to have unit variance across training set time windows, but were, due to their rectified nature, not transformed to have non-zero mean.

### Deep Learning Models

All deep learning models of the current study were implemented using the TensorFlow 1.12 library (Abadi et al., 2016) and executed in Python 3.6 using a desktop computer equipped with a Nvidia Titan V GPU. All architecture choices and hyperparameters were empirically selected on the basis of performance achieved on the training and validation sets; performance on test set data was never allowed to impact the design of deep learning models.

**FIGURE 1:**
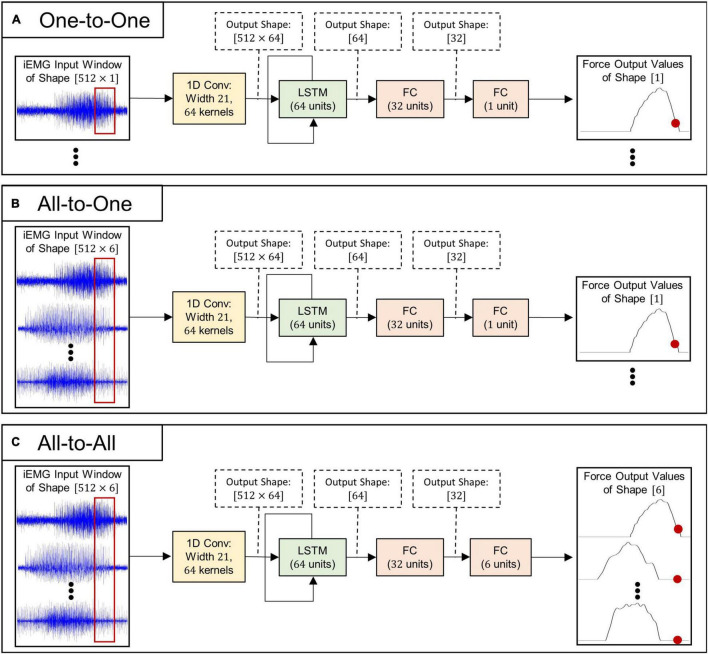
Schematic illustrations of deep learning algorithms used for myoelectric force regression: **(A)** model architecture used for the One-to-One regression strategy, **(B)** model architecture used for the All-to-One regression strategy, and **(C)** model architecture used for the All-to-All regression strategy.

Three separate strategies for regressing forces, each with a corresponding neural network architecture (all illustrated in Figure 1), were implemented in the current study:

1. **One-to-One.** Each individual force channel is estimated by a deep learning model processing a single matching iEMG channel. This approach requires six models in order to infer all output forces—one model per iEMG-force channel pair.
2. **All-to-One.** Each individual force channel is estimated by a deep learning model processing all available iEMG channels. This approach requires six models in order to infer all output forces—one model per force channel to be inferred.
3. **All-to-All.** A single LSTM model operates on all six iEMG channels and estimates all force channels simultaneously. This approach only requires that a single model is trained to infer all output forces.

As can be seen in Figure 1, the neural model architectures associated with the three above mentioned strategies are almost identical in structure: The input iEMG window is initially filtered by a 1D convolutional layer consisting of 64 filter kernels of size 21 with unit stride. Zero padding was used to keep the time dimension size of the output identical to the time dimension size of the input. Output feature maps of size 512=64 are subsequently fed into the central LSTM layer, whose output (of size *64*) at the final time step is fed into two consecutive fully connected layers. All convolutional-, LSTM-, and fully connected layers (except the last) are followed by leaky ReLU activation (Maas et al., 2013), layer normalization (Zhou and Yang, 2019), and dropout with probability 0.2. Notably, only the initial convolutional layer and the final fully connected layer differ in size between regression strategies; thus, the number of trainable parameters (and thus memory footprint and computational complexity) of the different model types (given in Table 3) is highly similar.

**TABLE 3:** Numbers of learnable parameters and inference times of models.

**Model**	**Number of parameters**	**Wall time of single inference**
One-to-One Linear	6×2	6×1ms
One-to-One LSTM	6×36801	6×54ms
All-to-One LSTM	6×43521	6×58ms
All-to-All LSTM	*43653*	62ms

Irrespective of regression strategy, all deep learning models were fitted to the training data in an identical manner using the AdamW algorithm (Loshchilov and Hutter, 2019) with learning rate η=10^−4^, β_1_ = 0.9, and β_2_ = 0.999 to iteratively minimize the mean squared error loss function. During training, a weight decay of λ=10^−9^ was applied. Training proceeded in minibatches of size 32 until the loss on the validation set had not decreased for 25 consecutive epochs or a total of 250 epochs had passed, whichever came first (i.e., a form of early stopping). For all model types, training was performed independently for each of the 16 recordings in the database. By design, for the One-to-One and All-to-One strategies, one model was trained per output force channel.

### Baseline Method

In addition to the deep learning processing pipeline described above, the current study included a conventional, amplitude-proportional linear control method in order to verify that the introduction of more computationally resource-intensive methods was warranted. A simple linear force regression algorithm was selected to represent the status quo for this purpose. The mean absolute values (MAV) feature (Hudgins et al., 1993) was computed channel-wise for each iEMG window in the training set and in the test set. Using the training set data, one univariate ordinary least squares (OLS) linear regression model was fitted to each iEMG channel to predict the concurrent force of its paired force channel. Consequently, this method is here referred to as *One-to-One linear regression*.

### Evaluation

The performance of the trained models was evaluated on the test set of iEMG time windows and corresponding ground truth force vectors by computing two standard offline regression metrics: the Root-Mean-Squared Error (RMSE) and the Variance Accounted For (VAF). Each computed scalar value represents the performance of a single regression method on a single recording session from the database.

The RMSE metric quantifies the normalized euclidean distance between the ground truth force values vector and the vector of force values produced by the regression model under consideration. The recording-wise RMSE is here symbolically defined in Equation 1:


(1) $$R\,M\,S\,E=\frac{1}{N}\,\sum_{n}^{N}\left(\frac{\sqrt{\frac{1}{C}\,\sum_{c=1}^{C}\left({\left({\hat{y}}_{n,c}-y_{n,c}\right)}^{2}\right)}}{y_{m\,a\,x}}\right)$$

where *N* is the total number of iEMG windows in the test set (variable across recording sessions) and *C* = 6 is the total number of force channels. *y*_*n,c*_ and ${\hat{y}}_{n,c}$ are the ground truth and estimated value, respectively, of the *c*th force channel associated with the *n*:th iEMG window and *y*_*max*_ is the maximum value of the regressand across the test set. Due to the fact that the RMSE metric increases as the discrepancy between the true and predicted value increases, a lower value represents better regression performance.

The VAF metric quantifies the proportion of variance of the ground truth that the trained model output accounts for. The recording-wise VAF metric is here symbolically defined in Equation 2:


(2) $$V\,A\,F=\frac{1}{C}\,\sum_{c}^{C}\left(1-\frac{V\,a\,r\,\left({\hat{\text{y}}}_{c}-{\text{y}}_{c}\right)}{V\,a\,r\,\left({\text{y}}_{c}\right)}\right)$$

**y**_*c*_ and ${\hat{\text{y}}}_{c}$ are aligned vectors of ground truth and estimated values, respectively, of the *c*th force channel. A higher VAF metric represents better regression performance.

### Statistics

Statistical computations were performed using functions provided with the SciPy library in Python. For all statistical analyses, differences at the α=0.05 level were considered significant. Initially, the non-gaussianity of all metrics and methods was tested with Shapiro–Wilk tests—as the gaussianity of RMSE metrics could not be rejected for any of the regression methods, a one-way repeated measures ANOVA was employed to detect any difference between methods. As a significant difference in RMSE between methods was detected in this way, *post hoc* analyses in the form of paired samples *t*-tests between all pairings of regression methods [$\left(\begin{array}{c} 4\\ 2 \end{array}\right)$ = 6 comparisons in total] were conducted. Furthermore, the arithmetic mean was selected as the summary statistic to represent the RMSE values achieved by each regression method over all recordings in the database. In contrast, the VAF metric was found to exhibit a significantly non-gaussian behavior for all regression methods. Consequently, a Friedman test was used to establish whether any difference between methods existed. Due to the non-gaussianity of the VAF metric, the median was selected as the summary statistic to represent the VAF values achieved by each regression method over all recordings in the database. As a significant difference in VAF between methods was detected by the Friedman test, *post hoc* analyses in the form of Wilcoxon signed-rank tests between all pairings of regression methods was conducted. For *post hoc* analyses performed on both metrics, acquired *p*-values were subject to Bonferroni correction for multiple comparisons; *p*-values are presented in corrected form throughout the Results section.

## Results

**FIGURE 2:**
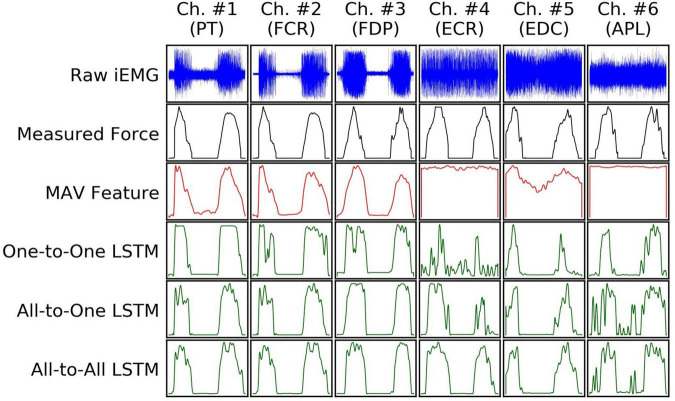
An example (from a random subject) of input iEMG presented to, and output force estimates produced by, all regression models, together with synchronous ground truth force measurements. Segments of iEMG channels (columns) and paired force channels shown here were selected from the task of the recording protocol in which they were maximally correlated; as such, the columns do not represent mutually concurrent time intervals in this illustration.

**FIGURE 3:**
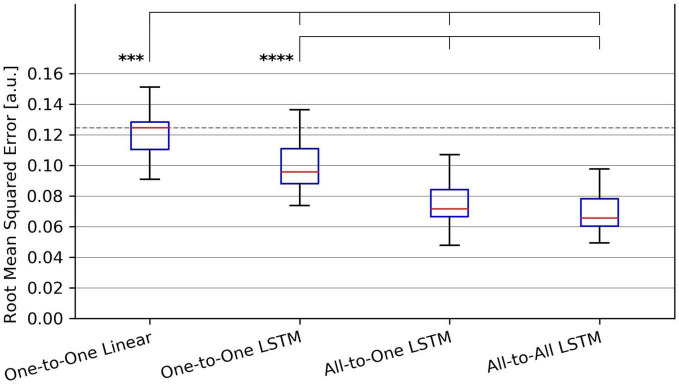
Tukey box plot of Root-Mean-Squared Error (RMSE) metrics achieved by all evaluated force regression methods. Whiskers extend 1.5 interquartile ranges below and above the first and third quartile, respectively. ***p < 0.001 and ****p < 0.0001.

**FIGURE 4:**
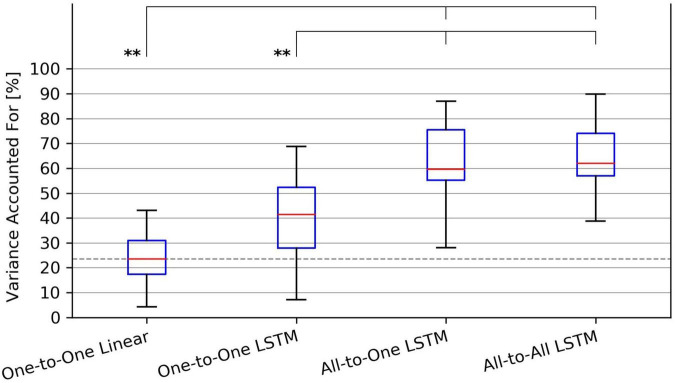
Tukey box plot of Variance Accounted For (VAF) metrics achieved by all evaluated force regression methods. Whiskers extend 1.5 interquartile ranges below and above the first and third quartile, respectively. **p < 0.01.

Examples of input and output signals produced by all regression methods are shown in Figure 2. RMSE and VAF values achieved by all models are summarized graphically in Figures 3, 4, respectively.

For the RMSE metric, one-way repeated measures ANOVA found a significant (*p* = 1.4⋅10^−9^) difference in performance between regression methods. As per *post hoc* paired samples *t*-tests, the decreases in mean between MAV-based linear regression (mean RMSE 0.123, SD 0.020) and the One-to-One strategy (mean RMSE 0.104, SD 0.018), the All-to-One strategy (mean RMSE 0.081, SD 0.018), and the All-to-All strategy (mean RMSE 0.077, SD 0.016) were 0.019 (*p* = 2.9⋅10^−4^), 0.042 (*p* = 1.1⋅10^−5^), and 0.046 (*p* = 1.0⋅10^−6^), respectively. Thus, all LSTM-based methods exhibited significantly better performance than the baseline method. Furthermore, both the All-to-One strategy and the All-to-All strategy significantly outperformed the One-to-One strategy, with differences in mean of 0.023 (*p* = 1.6⋅10^−6^) and 0.027 (*p* = 2.6⋅10^−8^), respectively. Lastly, a non-significant difference in mean of 0.004 (*p* = 2.2⋅10^−1^) was found between the All-to-One strategy and the All-to-All strategy. Significant differences in RMSE between methods as presented in this section are summarized in Table 4.

**TABLE 4:** Paired absolute differences in mean value of the RMSE metric across recording sessions (upper triangular part of table), and corresponding *p*-values (lower triangular part of table), separating regression methods.

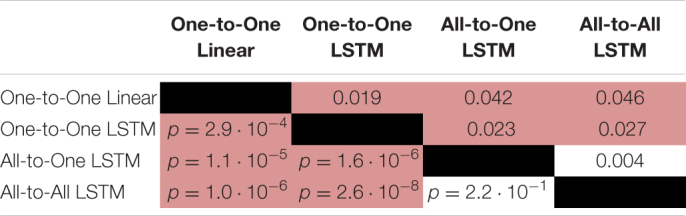

*Pairings exhibiting a significant difference at the α = 0.05 level are shaded red.*

For the VAF metric, a Friedman test found a significant (*p* = 1.9⋅10^−6^) difference in performance between regression methods. As per *post hoc* paired samples Wilcoxon signed rank tests, the increases in median between MAV-based linear regression (median VAF 21.5%) and the One-to-One strategy (median VAF 40.3%), the All-to-One strategy (median VAF 59.6%), and the All-to-All strategy (median VAF 60.9%) were 18.8% (*p* = 9.0⋅10^−2^), 38.0% (*p* = 3.8⋅10^−3^), and 39.4% (*p* = 4.6⋅10^−3^), respectively. Thus, all LSTM-based methods with the exception of the One-to-One strategy exhibited significantly better performance than the baseline method. Furthermore, both the All-to-One strategy and the All-to-All strategy significantly outperformed the One-to-One strategy, with differences in median of 19.2% (*p* = 5.6⋅10^−3^) and 20.6% (*p* = 2.6⋅10^−3^), respectively. Lastly, a non-significant difference in median of 1.3% (*p* = 1.0) was found between the All-to-One strategy and the All-to-All strategy. Significant differences in VAF between methods as presented in this section are summarized in Table 5.

**TABLE 5:** Paired absolute differences in median value of the VAF metric across recording sessions (upper triangular part of table), and corresponding *p*-values (lower triangular part of table), separating regression methods.

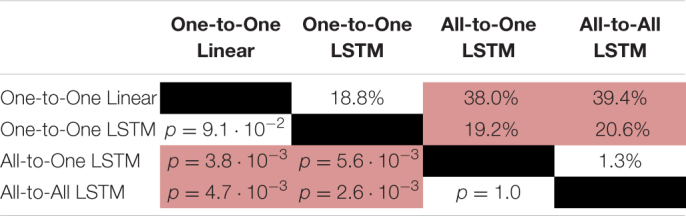

*Pairings exhibiting a significant difference at the α = 0.05 level are shaded red.*

## Discussion

The main aim of this study was to investigate the use of deep learning models based on the LSTM architecture to regress forces pertaining to multiple kinematic DoFs from concurrently acquired iEMG—specifically, the possibility of circumventing the need for hand-crafted signal features by letting such models map raw iEMG segments directly to regressand force values. From the obtained results, it was apparent that all proposed deep learning regression strategies outperformed the baseline One-to-One linear regression method in the sense of producing significantly lower mean RMSE values across recordings. Furthermore, two out of three deep learning methods were found to produce significantly better VAF metrics than the baseline. Together these findings lend credence to a view of end-to-end force estimation via deep learning methodology in general and via LSTMs in particular as a promising method to increase the accuracy of real-time proportional motor intent decoding through iEMG processing.

An additional aim was to evaluate differences in performance between the three examined deep learning regression strategies: estimating the force exerted by each DoF separately from a single iEMG channel originating from the active muscle (One-to-One), estimating each force channel separately but from all available iEMG channels (All-to-One), or directly estimating all force channels simultaneously from all available iEMG channels (All-to-All). For both computed performance metrics, it was found that the All-to-All and All-to-One strategies significantly outperformed the One-to-One strategy, indicating that information from muscles other than the prime mover increased the accuracy of force estimates. Two explanations of this finding are hypothesized here: First, aside from a single prime mover muscle, multiple synergist muscles can be causally implicated in the actuation of a single DoF. Relatedly, when one tries to selectively produce isometric force on a specific joint, other joints are usually stabilized by co-contraction, or even opposing contraction of the antagonist muscle. Measurements from such ancillary muscles could thus help regression models paint a more exhaustive picture of the biomechanical state of the arm when estimating forces. Second, even signals from muscles not mechanically involved in the current motion could in theory provide contextual information (of factors such as pose, fatigue, etc.) that correlate non-linearly with exerted force and, by extension, gives rise to better model performance.

From the perspective of conserving computational resources, it is promising that the All-to-All strategy performed at a level either higher than or indistinguishable from both of the other deep learning regression strategies; as only a single model is required to regress the forces exerted by all DoFs, both the computational complexity and memory footprint of this strategy are lower compared to the other approaches. This is of particular interest for prosthesis control—algorithms that would need to be implemented in an embedded processing environment, where computational resources are limited. Unfortunately, the resources required by the All-to-All LSTM model are still markedly higher than those of status quo feature-based linear control methods, even when considering the fact that no feature extraction step is necessary in the processing pipeline. The total number of parameters required to instantiate an All-to-All LSTM model was in this study 43,653 (see Table 3); assuming single-precision floating-point numbers are used, these require 175 kB to store in memory. Together with all intermittent activation maps (i.e., output volumes of layers) of the model requiring 538 kB to store, this represents a total memory footprint of approximately 713 kB—well within limits of existing embedded processors. As such, it is unlikely that memory footprint would be a limiting factor in realistic scenarios; the main bottleneck in this regard is instead likely the inference delay of the model. In principle, the highest acceptable inference delay is equal to the time between consecutive iEMG windows—with this delay, force values of windows are inferred at the same rate as that which they are sampled at. Whereas the inference delay of the All-to-All strategy (Table 3) narrowly falls below the window step size of 62.5 ms, values were measured from a model running on a GPU-equipped desktop computer. In an embedded application, the window duration could be decreased, and/or the window separation time could be increased, to reduce the real-time computational burden. However, for control delay not to become noticeable by the prosthesis user, the delay of the control system should fall below 300 ms (Englehart and Hudgins, 2003). With mechanical delays inherent to the prosthesis itself, this leaves approximately 125 ms available for algorithm-induced delays (Farrell and Weir, 2007). To circumvent these issues, a possibility is to let LSTM models operate continuously on iEMG samples as they are acquired instead of on a window-wise basis, as has been considered in previous studies using sEMG (Olsson et al., 2019). Nevertheless, it is apparent that future work that focuses on finding more computationally efficient LSTM regression architectures would be of value. As all algorithms considered in the current study were both trained and evaluated on a publicly available dataset, comparisons with alternative methods can be carried out transparently and straightforwardly.

Aside from the questions of computational complexity discussed above, a salient limitation of the approach taken in the current study is the necessity of regressand force values. Naturally, for amputee prosthesis users, acquisition of target force values prior to model training would not be possible. For the supervised learning approach to model training taken in the current study to be made applicable, some appropriate proxy regressand would thus have to substitute for forces measured at the level of the hand and wrist. A well-tried candidate solution is the use of mirrored training (Nielsen et al., 2011), whereby force measurements would be taken from the intact, contralateral hand while the amputee performs motions bilaterally. Another possibility is to ask the user to slowly increase and decrease the intensity of muscle contraction in accordance with some visual cue and subsequently use said cue as ground truth to be inferred from EMG, as has been investigated previously (Ameri et al., 2019).

The superior performance of deep learning methods in the current study is in line with findings from the general machine learning literature that indicate that signal representations automatically learned from data are oftentimes more informative than features designed manually toward the same end (Bengio et al., 2013). An intriguing research direction of specific interest for developing embedded, online motor decoding systems is that of reverse engineering the content of learned EMG features. If carried out successfully, this project could allow classical methods to enjoy some of the higher performance exhibited by deep learning methods without as high computational costs. Furthermore, for the purpose of leveraging the finding that information from multiple muscles improve performance, comparisons with sensor fusion techniques that allow for non-linear feature interactions (e.g., kernel regression; Bishop, 2006), but are less computationally demanding than recurrent neural networks, could be the focus of future work.

## Data Availability Statement

Publicly available datasets were analyzed in this study. These data can be found here: https://figshare.com/s/06f113bd74ecf6384729.

## Ethics Statement

The studies involving human participants were reviewed and approved by the Regional Ethics Review Board in Lund, Sweden. The patients/participants provided their written informed consent to participate in this study.

## Author Contributions

AO implemented the deep learning algorithms, analyzed the results, created the figures, and drafted the manuscript. NM, AB, and CA provided critical comments and finalized the manuscript for publication. All authors contributed to the design of the study.

## Conflict of Interest

The authors declare that the research was conducted in the absence of any commercial or financial relationships that could be construed as a potential conflict of interest.

## Publisher’s Note

All claims expressed in this article are solely those of the authors and do not necessarily represent those of their affiliated organizations, or those of the publisher, the editors and the reviewers. Any product that may be evaluated in this article, or claim that may be made by its manufacturer, is not guaranteed or endorsed by the publisher.
